# Fill the critical care discovery pipeline with *ICMx*!

**DOI:** 10.1186/s40635-020-00355-7

**Published:** 2020-11-09

**Authors:** Nicole P. Juffermans, Marcin Osuchowski, the ICMx Editorial board

**Affiliations:** 1 Department of Intensive Care, Laboratory of Experimental Intensive Care and Anesthesiology, OLVG Hospital, Amsterdam, The Netherlands; 2 Ludwig Boltzmann Institute for Experimental and Clinical Traumatology, AUVA Trauma Research Center, Vienna, Austria

*Intensive Care Medicine Experimental (ICMx)* was founded as an official journal of the European Society of Intensive Care Medicine (ESICM) community, in recognition that an improved understanding of the underlying pathophysiology of critical care syndromes is essential to advance clinical care for our patients. As a sister journal to *Intensive Care Medicine* (*ICM),* that focusses on publishing clinical research, the aim of *ICMx* is to offer a dedicated platform for publication of experimental oriented research. The need for such a platform is exemplified by a steadily increasing number of submitted manuscripts by researchers from all continents. In addition, the volume of articles downloaded from *ICMx* has been increasing annually; similar is true for the number of citations and postings on social media.

Currently, *ICMx* is shifting gears. In medicine, basic science refers to research that is not necessarily related to therapeutic strategies, whereas translational science refers to the translation of basic science findings into a development of potential therapeutic targets. With a newly appointed editor-in-chief and editorial board, *ICMx* will put a stronger focus on promoting translational research. More specifically, *ICMx* aims to publish research aspiring to: (1) advance understanding of mechanisms of critical illness; (2) support and/or enrich clinical trial design; (3) test novel treatment interventions and (4) develop and refine experimental models that are relevant to critical care conditions. This means that *ICMx* will publish experimental research as well as research performed in patients or with patients’ material. For experimental work, researchers are encouraged to provide a clear and executed vision of clinical relevance. The clinical studies would be well-executed earlier phase studies or studies with patient samples revealing biological insights that are likely to lead to further translational studies. The baseline criterion for manuscripts is that data contribute to the development of potential therapies and the refinement of existing treatments, as well as diagnostic and monitoring tools that improve critical care. The overall aim of our change in publishing policy is to bring discoveries and improved treatments and/or diagnostics closer to the bedside of our patients.

Since the founding of *ICMx*, the need for translational science to improve critical care has only become louder [1–3]. An apt example is the 2016 redefinition of sepsis as a “life-threatening organ dysfunction caused by a dysregulated host response to infection”. One of the important reasons for creation of the new sepsis definition was to refocus basic research so that it more robustly advances understanding of sepsis pathogenesis. Basic science research priorities have been formulated by the Surviving Sepsis Campaign Research Committee in 2018 [4] and expanded in April 2020 [5], providing numerous basic research questions with a high translational potential that urgently need to be answered. The same consideration applies to Acute Respiratory Distress Syndrome (ARDS). A prime example of success in translational critical care research has been the identification of specific phenotypes in sepsis and ARDS that can inform clinical trials design, moving into the era of personalized medicine.

We are only at the beginning of this approach. Personalizing mechanical ventilation is voiced as a top research priority. Its successful implementation requires an improved understanding of the complex ARDS biology and sepsis pathogenesis in order to more appropriately inform therapeutic discoveries [6].

How does *ICMx* stand out in bringing discoveries and potential novel treatments closer to the bedside? The editorial board of *ICMx* is the strong driving force in pursuing this goal. The board has been recently renewed, constituting a dedicated team of top-notch researchers. An important asset of the *ICMx* board is that members are a mix of both clinicians and basic scientists. Each member has a strong track record in a specific area of the critical care research. Together, they complementarily cover the full range of critical care pathobiology. A novel, regularly provided element in the updated modus operandi of *ICMx* will be thematic issues summarizing specific research questions with a high translational potential. There is a wide gap between successful pre-clinical interventions and many failed follow-up clinical applications. These thematic issues aim to improve the preclinical-to-clinical transition of knowledge. Furthermore, to create an active and dynamic dialogue with the readership and the research community at large, editorials will be invited which put recent findings into context and which describe a roadmap. And last but not least, *ICMx* is ambitious in obtaining an impact factor in the near future.

With a synergistic and highly competent editorial board, a strong position within the ESICM community and collaborative partnership with *ICM*, *ICMx* is uniquely positioned at the intersection of clinical and basic critical care science. We, the editorial board, wholeheartedly invite you not only to be its readers, but also to be part of *ICMx*’s growth and continued success by contributing your valuable research. Let us fill the discovery pipeline for critical care!

## Data Availability

Not applicable.
